# Primary Hyperparathyroidism: A Narrative Review of Diagnosis and Medical Management

**DOI:** 10.3390/jcm10081604

**Published:** 2021-04-09

**Authors:** Karel Dandurand, Dalal S. Ali, Aliya A. Khan

**Affiliations:** Division of Endocrinology and Metabolism, McMaster University, Hamilton, ON L8S 4L8, Canada; karel.dandurand@usherbrooke.ca (K.D.); d_alali@hotmail.com (D.S.A.)

**Keywords:** primary hyperparathyroidism, cinacalcet, bisphosphonates, denosumab, estrogen, raloxifene

## Abstract

Primary hyperparathyroidism (PHPT) is the most common cause of hypercalcemia in the outpatient setting. Symptomatic presentation includes non-specific signs and symptoms of hypercalcemia, skeletal fragility, nephrolithiasis and nephrocalcinosis. The majority of individuals present at an asymptomatic stage following routine biochemical screening, without any signs or symptoms of calcium or parathyroid hormone (PTH) excess or target organ damage. Indications for surgery have recently been revised as published in recent guidelines and consensus statements. Parathyroidectomy is advised in patients younger than 50 years old and in the presence of either significant hypercalcemia, impaired renal function, renal stones or osteoporosis. Surgery is always appropriate in suitable surgical candidates, however, medical management may be considered in those with mild asymptomatic disease, contraindications to surgery or failed previous surgical intervention. We summarized the optimal medical interventions available in the care of PHPT patients not undergoing parathyroidectomy. Calcium and vitamin D intake should be optimized. Antiresorptive therapy may be used for skeletal protection in patients with an increased fracture risk. Cinacalcet, a calcimimetic agent, has been shown to effectively lower serum calcium and PTH levels. The effect of medical treatment on the reduction in fracture risk is unknown and should be the focus of future research.

## 1. Introduction

**Figure 1:**
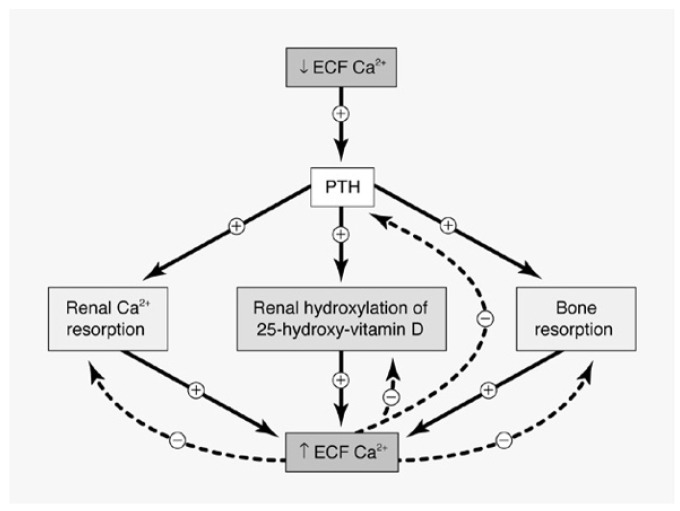
(Reproduced with permission from Khan et al., CMAJ 2000) Simplified representation of calcium homeostasis, with the regulation of serum calcium levels via feedback inhibition through the calcium receptor. ECF, extracellular; Ca, calcium; PTH, parathyroid hormone [8].

Primary hyperparathyroidism (PHPT) is a common endocrine disorder with an estimated prevalence of 0.86% in the United States [1,2] and represents the leading cause of hypercalcemia in the outpatient setting [3]. It results from the abnormal regulation and excessive release of parathyroid hormone (PTH) from one or more of the parathyroid glands. Increased levels of PTH lead to hypercalcemia through increased renal tubular calcium reabsorption, stimulation of osteoclast-mediated bone resorption and increased renal synthesis of 1.25 (OH)_2_D_3_, which in turn promotes enhanced intestinal calcium and phosphate absorption (Figure 1). Overt manifestations of PHPT have classically targeted the skeletal and renal systems, with patients traditionally presenting with reduced bone mineral density (BMD) or fragility fractures, as well as kidney stones, nephrocalcinosis or renal insufficiency [4]. However, the inclusion of serum calcium measurement in baseline metabolic panels has led to a gradual shift in the clinical presentation of PHPT in recent decades, from symptomatic disease to an asymptomatic presentation which is now the dominant phenotype in the Western world [5]. Approximately 70–80% of patients now present at an asymptomatic stage [6] and do not meet traditional criteria for surgery and may be followed with medical intervention if and as needed. Surgery is clearly the option of choice for those with symptomatic disease and may also be suitable for those with asymptomatic disease in the absence of comorbidities, as it is the only curative treatment strategy [7]. A conservative approach may also be warranted in patients who are not suitable surgical candidates or who do not wish to proceed with surgery or who have failed previous surgical intervention. Recent guidelines [7] and consensus statements [1] have provided recommendations on the evaluation, diagnosis and management of PHPT. This article summarizes the optimal medical management of patients with PHPT not undergoing parathyroidectomy.

## 2. Materials and Methods

In order to produce this narrative review, we carried out a literature search on MEDLINE, EMBASE and PubMed databases, including terms for PHPT in association with calcium, vitamin D and all included drugs discussed. All relevant articles published between 1 January 2000 and 31 December 2020 were reviewed in detail. Key articles published before 2000 were also included in the synthesis. Recovered entries were limited to data pertaining to human patients written in English.

## 3. Diagnosis

Primary hyperparathyroidism is diagnosed in the presence of hypercalcemia and an elevated or inappropriately normal PTH level [6]. It is essential to ensure that serum calcium is not fictitiously elevated, and as such, serum calcium corrected for albumin or an ionized calcium should be obtained.

The use of lithium and hydrochlorothiazide may lead to elevated serum calcium and PTH levels and should be discontinued 3 months prior to repeating the lab profile and confirming a diagnosis of PHPT. It is essential to exclude familial hypocalciuric hypercalcemia (FHH), a rare autosomal dominant disorder with three identified variants leading to the abnormal function of the CaSR, as surgery is not indicated in the presence of FHH. FHH1 is caused by an inactivating mutation in the *CaSR* gene itself and is the most common form as it comprises approximately 65% of FHH patients [9]. FHH2 accounts for <5% of FHH cases and is due to loss-of-function mutations in the *GNA11* gene that encodes the G-alpha subunit 11 (Gα11) protein, responsible for the CaSR downstream activation of phospholipase C [9]. FHH3 results from the inactivating mutation of the *AP2S1* gene that encodes the adaptor-protein 2 sigma subunit, a scaffolding protein that is essential in CaSR endocytosis, and this mutation is found in approximately 20% of FHH patients without *CaSR* mutations [9]. Calculating the calcium to creatinine clearance ratio (CCCR) is helpful in making the distinction between FHH and PHPT, as FHH is a state of relative hypocalciuria. The CCCR is <0.01 in 80% of FHH cases and a CCCR > 0.02 makes FHH less likely [9], and it is worth mentioning that a CCCR of 0.024 was recently reported in a patient with a molecular diagnosis confirming the presence of FHH [10]. Approximately 20% of individuals with FHH have a CCCR between 0.01 and 0.02 and can overlap with the values encountered in PHPT patients. In those cases, a positive family history of asymptomatic hypercalcemia, a personal or familial history of failed neck exploration and a history of hypercalcemia present in childhood or young adulthood are all in favor of a FHH diagnosis [9]. If the diagnosis of FHH cannot be excluded clinically, the DNA analysis of the *CaSR*, *GNa11* and *AP2S1* genes is warranted. It is also important to exclude other factors resulting in a low CCCR and these include renal impairment, vitamin D inadequacy or low calcium intake, as well as the use of thiazide diuretics which enhance renal calcium reabsorption [9]. Racial factors can also impact the CCCR, and it was reported that 44% of PHPT patients of African descent have calcium excretion <100 mg/day [11].

PHPT is the result of a solitary adenoma in 85–90% of cases, with multiglandular hyperplasia being present in approximately 15% of the cases, and parathyroid carcinoma is fortunately a very rare occurrence (<1%) [6]. Although PHPT predominantly affects women post-menopause, the incidence rates in males and females are approximately the same in the younger population under the age of 45 years. [12]. The incidence rate of PHPT peaks in women of 65–74 years of age, with an annual incidence of 99/100,000 persons-years compared to 17.2/100,000 persons–years for males of the same age range [12]. There also appears to be difference in the incidence of PHPT amongst different ethnic groups, with African American being more commonly affected, followed by Caucasians, with rates in Asians and Hispanics being lower than those seen in the Caucasian population [13].

PHPT may arise as part of a syndromic disorder, such as multiple endocrine neoplasia (MEN) 1, MEN2, MEN4 or hyperparathyroidism-jaw tumor (HPT-JT) syndrome, or present as an isolated non-syndromic endocrinopathy. Both forms may either occur as hereditary disease or as a non-familial, sporadic disease. Patients presenting with features or family history consistent with MEN or HPT-JT syndromes need further evaluation and gene analysis [14]. Genetic testing may also be warranted in certain patients with apparently isolated sporadic disease in the presence of features suggestive of an underlying genetic etiology, including young age at diagnosis, multiglandular disease, parathyroid carcinoma or atypical parathyroid adenoma, as approximately 10% of these patients have been shown to harbor de novo germline mutations causing PHPT [15].

## 4. Complications and Natural History

### 4.1. Skeletal Manifestations

Although the classical radiological features of osteitis fibrosa cystica (salt -and-pepper skull appearance, distal clavicle tapering, subperiosteal bone resorption, bone cysts and brown tumors) are now seldom encountered in developed countries, the skeleton remains a principal target of disease activity in PHPT. Bone mineral density (BMD) assessed by dual-energy X-ray absorptiometry (DXA) technology has demonstrated bone loss to be the most significant at the one third radial site [16], a site comprised of 99% cortical bone, with relative sparing of the lumbar spine, a site rich in trabecular bone [17]. However, the assessment of bone structure by two other modalities, HRpQCT and trabecular bone score (TBS), have shown alterations in bone microstructure in the trabecular compartment as well as the cortical compartment [18,19], and both indices correlate with fracture risk independent of BMD values [20]. These findings are consistent with the epidemiological and prospective cohort data demonstrating PHPT to be associated with an increased risk of both vertebral and non-vertebral fractures [21,22,23]. Post parathyroidectomy (PTX), improvements in the microarchitecture, geometry, cortical thickness, and estimated bone strength [24] translate into a significant decrease in the incidence of fractures, seen as early as one year after successful surgical intervention.

**Figure 2:**
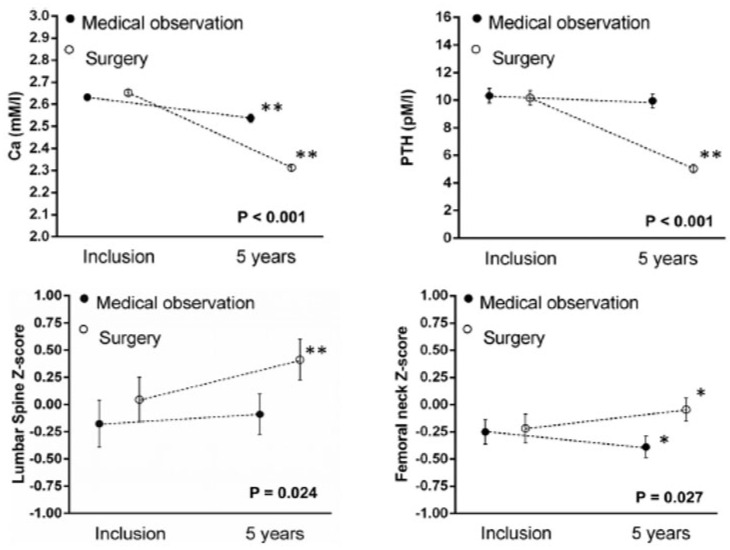
(Reproduced with permission from Lundstam et al., JCEM 2015) Biochemistry and bone mineral density (BMD). Serum calcium and PTH levels, and BMD Z-scores in lumbar spine (LS) and femoral neck (FN) at inclusion and after 5 years of follow-up in the two groups. P denotes a significant level for difference in change between groups. * *p* < 0.02 and ** *p* < 0.01 for within group change between inclusion and 5 years [30].

Long-term follow-up data of individuals with PHPT without intervention come from a 15-year observational study evaluating 57 patients, 86% of whom were initially asymptomatic [25]. Only serum calcium rose significantly beginning at year 13, whilst all other biochemical indices (PTH, ALP, urinary calcium) remained stable. Spine BMD remained stable while significant bone loss occurred at the distal radius and FN after 8 years of follow-up. Overall, 37% of asymptomatic patients developed new surgical indications over the study period. Shorter term randomized controlled trials (RCT) [26,27,28] of 1–2 years in duration have corroborated the stability of biochemical as well as BMD values in mild asymptomatic PHPT. The longest RCT conducted in mild asymptomatic PHPT showed a significant decrease in BMD at all sites except lumbar spine (LS) after 5 years (Figure 2), with a corresponding benefit of parathyroidectomy on BMD at all sites except for distal radius [29]. Five new vertebral fractures were found in the observation group and none in the surgical group, however, the results did not reach statistical significance due to the low incidence of new fractures [30]. Parathyroidectomy provides skeletal protection, however, the natural history of asymptomatic PHPT appears to indicate relative skeletal stability.

### 4.2. Renal Manifestations

Even though the prevalence of overt kidney stones has decreased dramatically from approximately 80% in older series to 15–20% in the modern era [31], it remains the most common complication of PHPT, and the prevalence of occult disease may be higher when patients are screened systematically [32]. Hypercalciuria appears to be a contributing factor in stone formation, with stone formers more likely to be hypercalciuric than non-stone formers [33]. However, since only a minority (less than one third) of hypercalciuric PHPT patients develop kidney stones, other urinary and non-urinary risk factors not yet clearly defined seem to be contributing to the development of stone disease [5]. After successful parathyroidectomy, the risk of recurrent stones decreases significantly, but unfortunately did not reach the values seen in the euparathyroid patient population and remains increased in comparison to a euparathyroid population [34].

Impaired kidney function has been described in PHPT, with up to 17% of asymptomatic patients presenting with an estimated glomerular filtration rate below 60 mL/min in one cross-sectional study of 294 patients [35], and it appears to be related to the severity and duration of hypercalcemia. Although the risk of progressive renal impairment in mild PHPT appears to be negligible over 2–3 years of observation [27], the demonstrated association of impaired kidney function (eGFR < 70 mL/min) with more severe declines in BMD support the surgical indication in PHPT patients with chronic kidney disease (CKD) [36]. Moreover, PTX was shown to prevent further decline in renal function in patients with preexisting renal impairment [37].

Nephrocalcinosis, the deposition of calcium in renal parenchyma, has been described in a small subset of PHPT patients (10.2% by spiral CT scan) [38], but to date the risk factors leading to nephrocalcinosis have not been clearly established and parathyroidectomy does not appear to have an impact on its presence or progression [39,40].

### 4.3. Non-Classical Manifestations of Other Organ Systems

Increased cardiovascular mortality is a well-established consequence of clinically overt PHPT [41,42,43]. Available data are more limited in the case of mild disease, but some studies suggest that this increase in mortality is also encountered in mild PHPT [23]. Hypertension has not been consistently reported to be associated with PHPT [44], and most importantly, an RCT assessing PTX versus observation on blood pressure control as a primary outcome did not demonstrate differences between the groups [45]. Decreased coronary flow reserve suggestive of altered coronary microvascular function has been suggested by studies in moderate to severely hypercalcemic PHPT patients [46,47], however, data on the risk of CAD in mild PHPT are still lacking.

PHPT appears to be associated with left ventricular hypertrophy [45], aortic valve calcification [48], increased aortic stiffness [49] and increased intimal media thickness [50] which may all contribute to the cardiovascular disease burden seen in PHPT. Unfortunately, parathyroidectomy has not consistently been demonstrated to lead to any improvement in these cardiac parameters. In the absence of more robust data, the presence of cardiovascular disease is still not considered a surgical indication in PHPT patients.

Many non-specific psychological and cognitive symptoms have been reported in PHPT patients, including fatigue, depression, anxiety, decreased working memory and concentration, as well as irritability. Given the inherent difficulty in assessing neuropsychological dysfunction and the observational nature of the majority of the related studies, it is difficult to draw firm conclusions regarding a possible causal relationship between PHPT and neuropsychological symptoms. The sum of the studies suggest an impaired quality of life in PHPT, however, improvement after PTX is inconsistent even amongst RCT using the same assessment tool [26,27,28]. Depression incidence appears to be increased in PHPT patients [23,51], however, three RCTs were not able to demonstrate any benefit post PTX [26,27,28]. Various aspects of cognition have been reported to be altered in PHPT, however, the specific domains affected varied across studies, and the only RCT assessing the effect of PTX on cognition failed to show a clinical benefit of surgical intervention [52].

Gastrointestinal symptoms have been frequently reported in PHPT patients, with constipation, heartburn, nausea and appetite loss being more common [4]. Although gastrinoma and associated peptic ulcer disease have been described to be more severe in MEN1 patients presenting with PHPT [53], a clear association between sporadic asymptomatic PHPT and PUD has yet to be proven, as studies have failed to consistently establish a correlation between PHPT and increased gastric acid secretion [54]. As pancreatitis seems to be related to the severity of the hypercalcemia rather than to the hyperparathyroidism itself, the incidence appears to be lower now that PHPT presents as a mild asymptomatic disease and therefore the occurrence of pancreatitis in asymptomatic PHPT is comparable to the general population [4].

## 5. General Measures, Calcium and Vitamin D

Adequate hydration should be maintained to avoid exacerbating hypercalcemia and precipitating hypercalcemic crisis. Thiazide diuretics should be avoided as they directly increase urinary calcium reabsorption in the distal renal tubule [55] Chronic lithium therapy may increase PTH by elevating the set point at which extracellular calcium suppresses PTH secretion, and therefore should be discontinued if the clinical situation permits.

Optimal calcium intake has been a debated subject. Through its effects on the cell surface CaSR, calcium inhibits parathyroid proliferation [56], with low intake stimulating PTH production and secretion. A 22-year-long prospective cohort study assessing the association between calcium intake and risk of PHPT in women was completed amongst more than 58,000 women of the Nurses’s Health Study 1 population [57]. The relative risk of PHPT was reduced (RR 0.56, 0.37–0.86, *p* = 0.009) in the group with the highest quintile of dietary calcium intake when compared to the lowest quintile, suggesting that chronically low calcium intake could contribute to further parathyroid grand proliferation and hyperplasia. A one-year open label study was conducted in 31 patients with asymptomatic PHPT in which those with a calcium intake less than 450 mg daily were given a 500 mg daily calcium supplement, whereas patients with intake above 450 mg were followed without intervention [58]. At 4 weeks into this study, there was a significant decrease in PTH levels in the group receiving the calcium supplement as well as an increase in FN T-score at one year, without a significant increase in serum or urinary calcium. In the light of these data, calcium intake in PHPT patients should not differ from those of the general population and should be in accordance with the Institute of Medicine guidelines for calcium intake [59].

The prevalence of vitamin D inadequacy is higher among PHPT patients than in the general population [60,61], as high PTH enhances the conversion of 25(OH)vitamin D into active 1.25(OH)_2_D [62] and also leads to vitamin D inactivation through enhanced hepatic metabolism [63]. Vitamin D insufficiency appears to be associated with the phenotypic severity of PHPT, as corroborated in a recent study conducted in 215 Caucasian Italian women with mild sporadic PHPT not taking vitamin D supplements [64]. There was a significant inverse correlation between lower quartiles of vitamin D levels and higher PTH and bone-specific alkaline phosphatase BSAP levels, however, no association was demonstrated between vitamin D levels and the incidence of fractures and nephrolithiasis, BMD values, or serum and urinary calcium. Other studies were able to demonstrate the association between low vitamin D status and increased bone turnover [65] as well as decreased BMD (FN, distal radius, whole body) [66]. Vitamin D insufficiency was also identified as an independent risk factor for post-operative hypocalcemia [67,68,69] and hungry bone syndrome [70]. Concerns regarding the effects of vitamin D repletion on further elevating serum calcium in PHPT have not been validated. A meta-analysis of 10 observational studies with a total of 340 vitamin D deficient patients with mild PHPT demonstrated significant increases in vitamin D levels, a mild decline in PTH levels and no changes in serum calcium, serum phosphate and urinary calcium with supplemental vitamin D [71]. There was, however, considerable heterogeneity in the supplemental regimen used, and as such, no specific repletion regimen can be recommended at this time. The only double-blinded RCT assessing vitamin D supplementation in PHPT was conducted in 40 patients, 26 weeks before parathyroidectomy, and used a 6-month course of cholecalciferol 2800 IU daily versus placebo. Results showed a significant increase in vitamin D levels, decreases in PTH levels (17%), increases in LS density (2.5%) and stable serum and urinary calcium [72]. The Consensus from the Fourth International Workshop on the Management of Asymptomatic PHPT recommends cautious vitamin D repletion, with supplemental doses of cholecalciferol ranging from 600 to 1000 IU daily, with close monitoring of serum and urinary calcium excretion, aiming for vitamin D levels above 50–75 nmol/L [73].

## 6. Pharmacologic Intervention

While patients with asymptomatic mild disease that do not meet surgical criteria may be followed conservatively, pharmacologic interventions may be considered in patients with osteoporosis or low BMD with progressive declines in BMD or in those who meet surgical criteria for interventions but are not able to proceed in the presence of comorbidities precluding parathyroidectomy, who refuse surgery or in those who failed surgery. It may also be an option for those with familial PHPT and persistent or recurrent hypercalcemia following parathyroidectomy.

### 6.1. Estrogen

Only one RCT evaluated the long-term effect of hormone replacement therapy (HRT) on BMD in a population of 42 post-menopausal women with mild asymptomatic PHPT [74]. The 4-year extension study [75] conducted in 23 patients of the original cohort showed that women treated with conjugated equine estrogen 0.625 mg plus medroxyprogesterone acetate 5 mg daily had increased BMD compared to placebo (4.6% at total body, 7.5% at LS, 7.4% at FN, 8.2% at femoral trochanter, and 7.0% at forearm). Serum calcium was stable in year 1 and 2 of the original study but was slightly lower in the 4 years of the extension. PTH remained stable and bone turnover markers (BSAP and urine hydroxyprolin excretion) decreased in the treatment group, while there was an initial reduction in urinary calcium excretion that was not maintained at 4 years. The incidence of fractures in this study was too low to evaluate an effect on fracture risk.

In post-menopausal women with mild asymptomatic disease, HRT has been shown to increase BMD and may be a valuable option for skeletal protection.

### 6.2. SERMS

A beneficial effect of raloxifene at LS and FN was first suggested in a short observational study of three post-menopausal women with mild asymptomatic PHPT after 12 months of active treatment [76]. A subsequent 12 months RCT compared the efficacy of raloxifene with alendronate in 24 PHPT women with osteoporosis. It demonstrated the beneficial effect of raloxifene at the LS but was not able to replicate the expected benefit at the FN or radial skeletal sites [77]. A shorter 8-week RCT was conducted in 18 post-menopausal women with asymptomatic PHPT to assess the effects of raloxifene on serum calcium and bone turnover markers and was able to demonstrate a significant decrease in serum calcium, osteocalcin and N-telopeptide, while PTH, ALP and urinary calcium excretion remained stable [78]. This will require further evaluation in a more robust study population.

SERMS may be of value in preventing bone loss at the lumbar spine in post-menopausal women with PHPT who are not at a high risk of fracture.

### 6.3. Aminobisphosphonates

A recent systematic review evaluated the efficacy of different drug classes, including bisphosphonates, in the management of PHPT [79]. Seven studies reported on the use of pamidronate, but all were of short-term duration (single infusion to a few weeks), so although there was a significant reduction in serum calcium within a few days of treatment (mean change 0.31+/−0.034 mmol/L), there were no data on BMD effects. Five out of the seven studies showed transient increases in PTH levels, as well as decreased bone turnover markers (urinary calcium, ALP, osteocalcin, urinary hydroxyproline).

**Figure 3:**
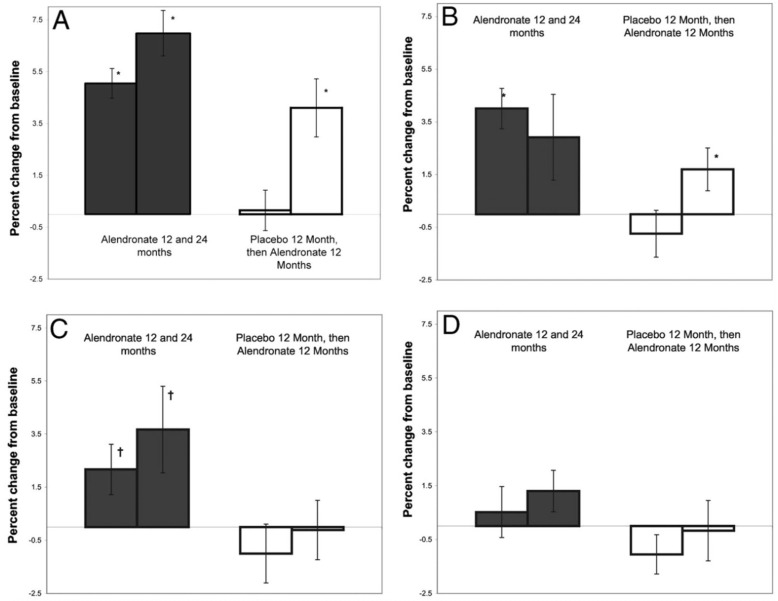
(Reproduced with permission from Khan et al. JCEM 2004) Effect of Alendronate on LS (**A**); TH (**B**); FN (**C**); and one third distal radius (**D**). * Significantly higher than baseline (*p* < 0.001), † significantly higher than baseline (*p* < 0.05) [80].

Alendronate remains the most widely investigated bisphosphonates, with 12 studies reporting on its use in PHPT, the majority of which had long-term data of ≥48 weeks, whilst seven of them used alendronate 10 mg once daily. The long-term decrease in serum calcium appears to be very marginal with alendronate (average 0.07+/−0.05 mmol/L), however, most of the studies reported a significant initial decrease that lasted for about 6 months before increasing back towards baseline values, with PTH levels that were correspondingly higher during this initial phase. All long-term studies reported increased BMD at the spine and hip with stability at the distal radius, a site rich in cortical bone. The largest double-blinded RCT evaluating alendronate treatment of 10 mg daily compared to placebo for one year was followed by a crossover period in which all patients received active drug treatment for another 12 months. This was conducted in 44 asymptomatic PHPT patients who either did not meet surgical criteria or declined surgery [80]. At the end of this study, BMD had significantly increased from the baseline to the LS, FN and total hip (TH) in the arm treated with alendronate for 24 months (Figure 3). In the placebo group, BMD was stable at all sites at 12 months and showed significant increases in LS and TH after 12 months of crossover to alendronate. Alendronate use was associated with rapid and marked reductions in bone turnover markers (urinary NTX, BSAP), while serum and urine calcium as well as PTH remained stable.

Risedronate was only evaluated in two studies, one short-term of 7 days that showed a small but significant decrease in serum calcium as well as a corresponding increase in PTH [81], another open label prospective study of 24 months duration that did not demonstrate any benefit of risedronate on BMD assessed by DXA or bone mineral content by pQCT [82].

Aminobisphosphonates have been shown to modestly increase spine and hip BMD in mild PHPT [83]. Bisphosphonates have not been shown to decrease serum calcium. Therapy with aminobisphosphonates may be considered for skeletal protection in patients with osteoporosis not proceeding with surgery.

### 6.4. Denosumab

Denosumab was first demonstrated to have a beneficial effect on skeletal protection in 25 women with PHPT-associated osteoporosis in a retrospective longitudinal study conducted in 2018 [84]. After 24 months of denosumab therapy, there was a significant decrease in ALP levels and a significant improvement in BMD at the LS, FN and TH sites. These results were supported by a randomized single-center double-blinded trial conducted in 46 PHPT patients which assessed the efficacy of combinations of cinacalcet + denosumab, versus denosumab + placebo, versus placebo alone [85]. At one year, there was a significant increase in BMD in denosumab-treated patients, irrespective of cinacalcet use, at LS (6.9%), TH (4.1%) and femoral neck (3.8%), but unfortunately the change at the distal radius did not reach significance. A recent retrospective longitudinal study aimed to evaluate the effects of denosumab compared to parathyroidectomy on serum calcium levels, renal function and bone turnover in osteoporotic PHPT patients [86]. Although the denosumab-treated group (*n* = 19) was older and with milder disease than the surgical group (*n* = 19), there was a significant increase in BMD at LS (6.0+/−0.8%), TH (3.7%+/−1.0%) and FN (4.3+/−1.5%). Serum calcium and creatinine levels remained stable in the denosumab group while they decreased, as expected, after parathyroidectomy.

Denosumab appears to provide skeletal protection at the spine and hip sites in patients with PHPT and has not been shown to lower serum calcium.

### 6.5. Cinacalcet

**Figure 4:**
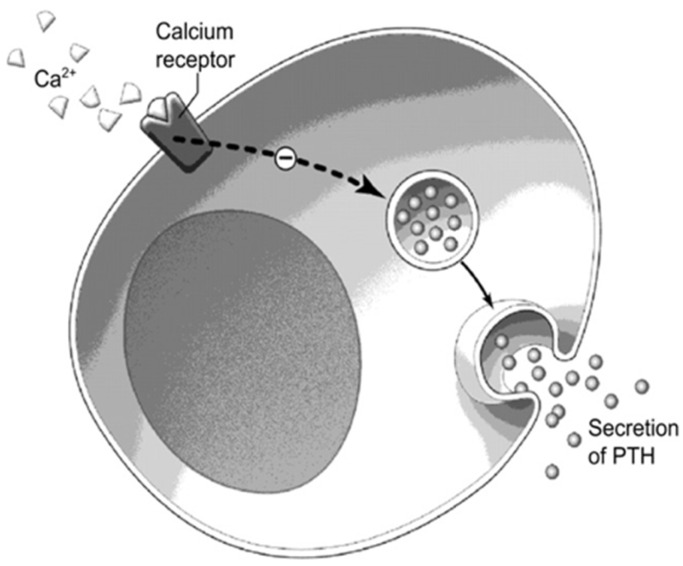
(Reproduced with permission from Khan et al., CMAJ 2000) Schematic illustration of calcium binding to the calcium receptor at the parathyroid cell and inhibiting PTH secretion. Ca, calcium; PTH, parathyroid hormone [8].

Calcimimetic agents increase the sensitivity of the CaSR to activation by extracellular calcium (Figure 4), in turn leading to the decreased synthesis and secretion of PTH, therefore lowering serum calcium. Cinacalcet is now approved in Canada and the USA for the treatment of PHPT patients unable to proceed with parathyroidectomy, as well as for the treatment of secondary hyperparathyroidism in dialysis patients and parathyroid cancer.

**Figure 5:**
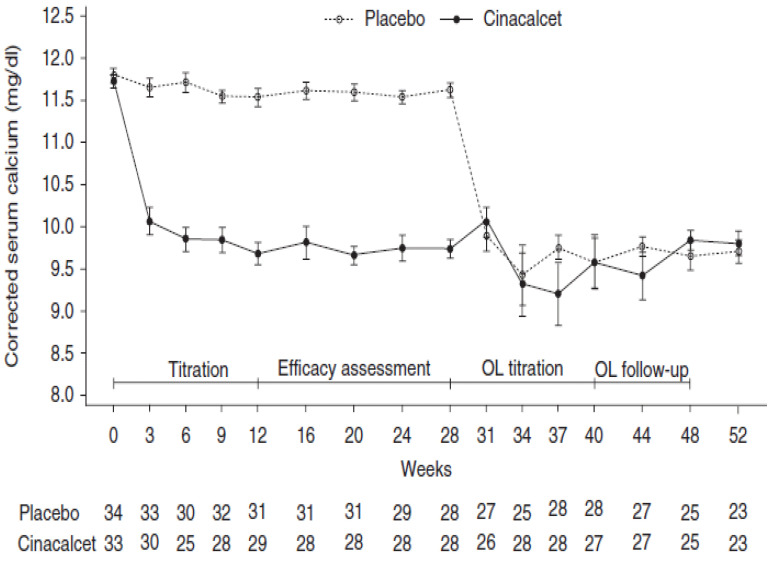
(Reproduced with permission from Khan et al. EJE 2015) Mean corrected serum calcium over time. OL: open label [87].

The first multicenter, international, phase 3 RCT reported on the use of cinacalcet in patients with moderate PHPT who met surgical criteria but were unable to undergo parathyroidectomy. This study was conducted in 67 subjects randomized (1:1) to cinacalcet or placebo for the first 28 weeks of the study, followed by an open label extension phase during which all patients received cinacalcet [87]. Cinacalcet was started at 30 mg twice daily with dose titration aiming to maintain normocalcemia; the median dose used was 60.2 mg/day. In the cinacalcet-treated group, serum calcium normalized (<2.575 mmol/L) in 75.8% of patients (0% in placebo group), PTH decreased by 23.8% (1.1% in placebo group) and phosphate level increased from a mean of 0.665 to 0.885 mmol/L (Figure 5). A recent systematic review and meta regression study reported on 28 articles, evaluating the effects of cinacalcet in PHPT. The majority of the studies were prospective and retrospective cohort studies. Four RCTs against placebo were also included, describing the use and efficacy of cinacalcet in a total of 722 PHPT patients [88]. Meta-regression reported a normalization rate of serum calcium of 90% (CI 0.82–0.96), with a mean reduction in calcium levels of 0.412 mmol/L (CI 0.343–0.481 mmol/L) from baseline. PTH levels normalized in only 10% of cases (CI 0.02–0.23), with a pooled analysis of 166 patients demonstrating a significant decrease in PTH levels of 26.796 pg/mL (CI −39.647 to −13.945 pg/mL, *p* < 0.001). Interestingly, a larger mean reduction in serum calcium was observed when initial calcium level was >3.0 mmol/L. Phosphate level was reported to rise significantly by 0.160 mmol/L (CI 0.400–0.596 mmol/L). The main side effects reported were mild-to-moderate gastrointestinal symptoms (nausea or vomiting in 23%), and only 3% of reported hypocalcemia cases. This meta regression did not report on the BMD effects of cinacalcet. The longest double blinded multicenter RCT included in this systematic review assessed the ability of cinacalcet to achieve long-term reduction in serum calcium and PTH as primary endpoints. It also explored the effect of cinacalcet on BMD and bone turnover in 78 PHPT patients over 52 weeks [89]. Normocalcemia was achieved in 73% of patients within the first 2 weeks of therapy and was maintained over the entire study period. There was no difference in spine BMD at 52 weeks, and in a 4.5-year cross-over open label extension study conducted in 45 patients of the original cohort all given cinacalcet, there was no significant change in BMD at LS, hip or distal radius [90]. Similar efficacy of cinacalcet on serum calcium normalization was reported in patients with intractable PHPT [91], symptomatic severe hypercalcemia [92] and parathyroid carcinoma [93,94], suggesting significant benefit across a broad spectrum of disease severity.

Cinacalcet has been shown to lower PTH and serum calcium and may be a valuable option in the presence of significant hypercalcemia due to PHPT if surgery is not an option. Cinacalcet has not been shown to offer skeletal protection. Combination therapy with cinacalcet and bisphosphonates has been evaluated [95,96] and provides skeletal protection with the lowering of serum calcium.

## 7. Conclusions

Parathyroidectomy remains the mainstay of treatment for primary hyperparathyroidism as it is the only curative option which can normalize parathyroid function and result in improvements in BMD and fracture risk [22,24,97]. It has also been shown to reduce the risk of renal stones [34] and preserve renal function [37]. However, for patients who do not meet the criteria for surgical intervention or for whom surgery may not be desirable or possible, targeted medical intervention is now available. Patients not proceeding to parathyroidectomy must be closely followed with regular biochemical and BMD assessment. Calcium intake should not be restricted and vitamin D inadequacy, if present, should be adequately corrected aiming for levels above 50 to 75 nmol/L. Antiresorptives offer skeletal protection, whereas cinacalcet should be reserved to effectively control hypercalcemia. We now have a better understanding of the utility of medical intervention and impact on BMD and biochemical profile. We still do not have data documenting an impact on fracture risk with medical intervention. This should be the focus of future research.
